# High-risk Allele for Herpes Labialis Severity at the IFNL3/4 Locus is Associated With Vestibular Neuritis

**DOI:** 10.3389/fneur.2020.570638

**Published:** 2020-10-08

**Authors:** Dan Rujescu, Marko Herrling, Annette M. Hartmann, Stephan Maul, Ina Giegling, Bettina Konte, Michael Strupp

**Affiliations:** ^1^Department of Psychiatry, Psychotherapy and Psychosomatics, Martin-Luther-University Halle-Wittenberg, Halle (Saale), Germany; ^2^German Center for Vertigo and Balance Disorders, University Hospital Munich, Munich, Germany; ^3^Department of Neurology, University Hospital Munich, Munich, Germany

**Keywords:** vestibular neuritis, HSV-1, IFNL3, IFNL4, vertigo

## Abstract

**Objective:** Vestibular neuritis (VN) is a peripheral vestibular disorder leading to a sudden loss of unilateral vestibular function. Although the underlying etiological mechanisms for disease development are not yet known, there is evidence that a latent infection with herpes simplex virus type 1 (HSV-1) might be involved. The polymorphism rs12979860 has been associated with the severity of recurrent herpes labialis and hepatitis C virus (HCV) clearance and treatment outcome and is located within the first intron of the IFNL4 gene on chromosome 19.q13.2. This case control study was conducted to evaluate the association of rs12979860 with VN occurrence.

**Methods:** DNA was extracted from EDTA blood of 151 VN patients and 1,775 healthy controls. Genotyping of rs12979860 was performed using iPLEX and MassARRAY Matrix Assisted Laser Desorption Ionization—Time of Flight (MALDI-TOF) mass spectrometry. For association analyses, an additive, dominant and recessive logistic regression model was calculated, using age and sex as covariates.

**Results:** A significant association of rs12979860 with VN was obtained for the additive [OR = 1.51 (1.18–1.92); *p* = 9.23 × 10^−4^] and dominant models [OR = 2.15 (1.48–3.13); *p* = 5.86 × 10^−5^], with the T allele being more frequent in the VN group.

**Conclusion:** By detecting a significant association of the rs12979860-T risk allele for herpes labialis severity with susceptibility to VN, this study gives further indirect evidence for an involvement of HSV-1 in VN pathology, thereby strengthening the virus hypothesis.

## Introduction

Vestibular neuritis (VN) is characterized by an acute onset of sustained spinning vertigo, oscillopsia, postural imbalance, nausea and vomiting due to a sudden loss of unilateral vestibular function. The estimated incidence of VN ranges between 3.5 and 15.5 per 100,000 subjects (1, 2) with a recurrence rate of about 2% (3) to 10.7% (4).

The mechanisms contributing to the development of VN have not yet been identified. According to the leading virus hypothesis, VN is caused by reactivation of a latent herpes simplex virus type 1 (HSV-1) infection, which leads to an accumulation of HSV-1 DNA and latency associated transcript (LAT) in cells hosting the virus (5–8). The virus hypothesis is also supported by a mouse model in which vestibular dysfunction and vestibular ganglion infection were induced after inoculation of HSV-1 and 2 (9), as well as by the observation of elevated acute phase proteins in the blood and an elevated percentage of CD40-positive peripheral blood mononuclear cells in VN patients (10, 11). The latter is associated with thrombotic events, so that proinflammatory processes and the associated increased expression of CD40 on monocytes and macrophages may lead to microvascular occlusion via aggregation of platelets with these cells (11, 12). In contrast to other complex diseases such as schizophrenia (13), little is known about the genetic factors underlying vestibular disorders. For VN, a genome-wide association study (GWAS) conducted in 151 VN patients and 2,609 healthy controls was recently published, with three genome-wide significantly associated loci with a link to viral replication (14). In addition to the virus hypothesis, immune-mediated processes were assumed to cause VN, since an unbalanced CD4/CD8 quotient was found in VN patients, which is similar to the findings in patients with multiple sclerosis [for review see (15)]. However, there has been no further progress in this area in recent years.

In a study by Griffiths et al. (16) an association of the single nucleotide polymorphism (SNP) rs12979860 with severity and recurrence rate of herpes labialis was observed, in which a dose-dependent effect of the T allele was determined. This is in line with studies conducted in patients with hepatitis C virus (HCV) infections. In a GWAS on HCV infected individuals, T allele carriers showed a significantly higher rate of chronic persistence of the virus, whereas C allele carriers showed a higher incidence of spontaneous virus clearance (17). In addition, the T allele was associated with non-response to antiviral therapy with pegylated interferon λ (IFN- λ) and ribavirin (18, 19) and also with decreased *IFNL3* mRNA levels (20). Interestingly, a reduced INF- λ response was also observed in patients with recurrent herpes labialis caused by HSV-1 compared to seropositive controls without recurrences (21).

The SNP rs12979860 is localized on chromosome 19q13.2 in a region containing the *IFNL* genes and was initially assigned to the promotor region of *IFNL3*. In fact, it is also located in the first intronic region of the *IFNL4* (pseudo)gene. Transcription of this gene depends on the dinucleotide variant rs368234815 located in the first exon of *IFNL4*. While TT renders IFNL4 to a pseudogene, the ΔG variant leads to a frameshift that generates an open reading frame and enables protein expression (22). The ΔG allele of rs368234815 and the T allele of rs12979860 are in strong linkage disequilibrium (LD) in European and Asian ancestries (*r*^2^ = 0.9, European, *r*^2^ = 1.0, Asian) (22), but considerably less in African populations (*r*^2^ ~ 0.7) (23).

IFN- λs (or type III IFNs) mediate antiviral, antibacterial and antifungal effects, with the four previously known INF- λ ligands (IFN- λ1-4) having different affinities to their receptor (IFNLR), which is a heterodimer consisting of an a- (IL28RA) and a b-subunit (IL10RB) showing a highly variable cell type specific expression pattern [for review see (24)]. Similar to type I IFNs (IFN-α and β), IFN- λs are able to inhibit replication of several virus types, e.g., HSV-1 (25), HSV-2 (26), and HCV (27). However, IFN- λ4 differs from the other type III IFNs in its kinetics, its earlier release during viral infections and its ability to induce the expression of genes that negatively regulate IFN response (28). Interestingly, it has been shown that expression of *IFNL4* mRNA *per se* was associated with a reduced HCV clearance and a poorer response to antiviral therapy. Thus, a possible functional relationship between the described polymorphisms and the associated phenotypes was proposed (20, 22).

Since reactivation of a latent HSV-1 infection is discussed as a cause for VN and severity and recurrence rates of herpes labialis caused by HSV-1 infection are associated with rs12979860, a variation localized in the IFNL4 gene, an influence of this variation on VN pathology could be hypothesized. Therefore, a case-control study investigating the association of rs12979860 with VN was conducted.

## Materials and Methods

### Study Population

Participants of European ancestry included in the study were recruited from the Greater Munich area (Germany) and clinical interviews were conducted at the German Center for Vertigo and Balance disorders, the Department of Neurology (patients) and the Institute for Psychiatry (controls), at the Ludwig-Maximilians-University Munich from 1997 to 2016. Detailed medical histories of the participants and their first-degree relatives were assessed using a semi-structured interview.

#### Patients

The cohort of VN patients has already been described elsewhere (14). In brief, 151 patients who met the diagnostic criteria for unilateral VN (29) were included in the study. The diagnosis was based on the patient's medical history, the clinical examination and, in ambiguous cases, further examinations such as MRI and caloric testing. The key symptoms, all of which had to last for at least 72 h, were a history of acute/subacute onset of sustained spinning vertigo, oscillopsia, gait imbalance and nausea or vomiting. Horizontal-torsional peripheral vestibular spontaneous nystagmus toward the unaffected ear, a pathological head impulse test (HIT) toward the affected side, suppressed by visual fixation, and postural imbalance with Romberg fall toward the affected ear were required in the neurological examination. In addition, caloric testing had to show a hypo- or unresponsiveness of the affected horizontal canal with an >25% asymmetry between both sides. A video HIT was performed in all patients with a gain of the vestibulo-ocular reflex < 0.7 required on the affected side. Patients with any evidence for other peripheral and central vestibular or ocular motor disorders (see below) were excluded. In addition, a history of acute hearing loss, brainstem or cerebellar symptoms as well as clinical evidence for a central ocular motor lesion, i.e., skew-deviation, saccadic smooth pursuit, gaze-evoked nystagmus, normal head-impulse test led to exclusion from the study (30). Patients with recurrent VN as well as with chronic hepatitis B, C or HIV infection were not included.

#### Healthy Controls

DNA samples from 1,775 unrelated healthy subjects of European ancestry were taken from the Phenomics and Genomics Sample (PAGES), which is comprised of ~3,000 controls, 1,000 schizophrenia patients and 300 individuals with other diagnoses. The Structured Clinical Interview (SCID I and SCID II) and the Family Assessment Module were performed on all healthy volunteers (31–33). Individuals with a history of VN (self-reported) as well as those, suffering from neurological or psychiatric diseases or with first degree relatives with these disorders were excluded.

### Informed Consent

Informed consent was obtained from all participants, and the study was approved by the Ethics Committee of the Ludwig-Maximilians-University Munich and carried out in accordance to the Declarations of Helsinki.

### Genotyping

Genomic DNA was extracted from peripheral white blood cells using the QIAamp DNA Maxi Kit (Qiagen, Hilden, Germany), according to the manufacturer's protocol, and dissolved in nuclease free water. Using picogreen (Invitrogen, Karlsruhe, Germany) the DNA concentration was measured and subsequently adjusted to 50 ng/μl.

Genotyping of rs12979860 was performed using the MassARRAY platform (Sequenom, San Diego, CA) as described previously (34) with minor adaptations. PCR primer and extension primer were designed using the Assay Designer 4.0 software (Sequenom, San Diego, CA). 12.5 ng of genomic DNA, 0.5 mM dNTP (ABgene, Hamburg, Germany), 100 nM PCR primer (forward: ACGTTGGATGAGCGCGGAGTGCAATTCAAC, reverse: ACGTTGGATGTCGTGCCTGTCGTGTACTGA) (Metabion, Martinsried, Germany), 1.625 mM MgCl_2_, and 0.5 U HotStar Taq-polymerase (Qiagen, Hilden, Germany) were used to perform the initial PCR (initial denaturation at 95°C for 5 min; 45 cycles with 20 s at 95°C, 30 s at 56°C, 1 min at 72°C; final elongation 3 min at 72°C). Following a shrimp alkaline phosphatase treatment, the iPLEX reaction mix containing the extension primer (TGCAATTCAACCCTGGTTC) (Metabion, Martinsried, Germany), was added. PCR reaction was carried out with the following parameters: initial denaturation at 95°C for 30 s, 40 cycles with 5 s at 94°C and 5 cycles at 52°C for 5 s, 80°C for 5 s, final elongation at 72°C for 3 min. After desalting the extension products, samples were spotted on SpecroCHIPs GenII (Sequenom, San Diego, CA) and analyzed with the MassARRAY MALDI-TOF mass spectrometer. Allele-specific extension products and resulting genotypes were identified by Typer 3.4 Software (Sequenom, San Diego, CA).

### Quality Control

The following quality criteria were applied to confirm reliable genotypes: compliance with the Hardy-Weinberg equilibrium (HWE) per genotyping unit containing 376 samples, in the control and combined sample (*p* ≥ 0.05), sample callrate > 80% and SNP callrate > 95%. Deviation from the HWE was accepted for cases, if controls on the same genotyping unit were in compliance with the HWE. In addition, the allele frequency of the genotyped control group was compared with genetic datasets of populations from Europe, provided by 1,000 Genomes Project Phase 3 on Ensembl (35) and showed a similar distribution (T-allele: 33.9% in the genotyped compared to 30.9% in the European sample).

### Statistical Analyses

Group differences in sex and age were calculated using exact Fisher's exact test and Wilcoxon rank sum test. Deviations of the genotype frequency from HWE in the patient, control and combined samples were analyzed using Pearson's chi-squared test. After quality inspection, a logistic regression was calculated using plink 1.7 (36, 37). Additive (additive effect of increasing amounts of the minor allele), dominant (homozygous major allele carriers vs. carriers of the minor allele) and recessive genotype models (homozygous minor allele carriers vs. carriers of the major allele) corrected for age and sex were used to check for an association of rs12979860 genotypes with VN. An a posteriori power calculation was performed using the software Quanto (38) with the following parameters: unmatched case-control study (1:11.75), minor allele frequency of 0.35 (total sample), additive and dominant inheritance models, incidence rate of 0.0155% (as no data on prevalence rates are available) and significance level of *p* < 0.05.

## Results

In this study 151 patients with unilateral VN and 1,775 healthy controls were included in the analysis. The mean age of patients with VN (64 females, 42%) was 55.4 ± 14.8 years and the mean age of controls (910 females, 51%) was 55.1 ± 13.4 years, sex showing a slight statistical difference between groups (sex: *p* = 0.04, age: *p* = 0.91).

The T allele of rs12979860 located on chromosome 19q13.2 within the first intron of *IFNL4*, depicted the minor allele with a frequency of 43.4% in cases and 33.9% in healthy controls (Table 1). The control group and the combined sample were in Hardy-Weinberg equilibrium (controls: χ^2^ = 0.05, *p* = 0.82 and combined sample: χ^2^ = 0.88, *p* = 0.35), whereas the case group showed a slight deviation (χ^2^ = 7.77, *p* = 0.005).

**Table 1:** Allele and genotype frequencies of rs12979860.

	**Allele**		**Genotype**		
**Sample**	**C *n* (%)**	**T *n* (%)**	**CC *n* (%)**	**CT *n* (%)**	**TT *n* (%)**
VN	171 (56.6)	131 (43.4)	40 (26.4)	91 (60.2)	20 (13.2)
Controls	2,346 (66.3)	1,204 (33.9)	773 (43.5)	800 (45.1)	202 (11.4)
Combined	2,517 (65.3)	1,335 (34.7)	813 (42.2)	891 (46.3)	222 (11.5)

For association analyses in this case-control study, additive, recessive and dominant logistic regression models corrected for age and sex were calculated. The results are summarized in Table 2. The additive model revealed a significant association of rs12979860 with VN (*p* = 9.23 × 10^−4^, OR = 1.51, 95% CI = 1.18–1.92), with the T allele being more prevalent in the VN group compared to the control group. A significant result was also found in the calculation of the dominant model (carriers of the minor allele TT + CT vs. homozygote carriers of the major allele CC). This resulted in an OR of 2.15 (95% CI = 1.48–3.13) for carriers of the T allele and compared to healthy controls, the proportion of T allele carriers was higher in the VN group. Using the recessive model, no significant association was found. Based on the results of our study, a power calculation was performed, which yielded a statistical power of 92.17% for the additive model (OR = 1.51) and 98.60% for the dominant model (OR = 2.15).

**Table 2:** Logistic regression analysis.

**Model**	***P*-value**	**OR (95% CI)**	**SE**
Additive	9.23 × 10^−4^	1.51 (1.18–1.92)	0.12
Dominant	5.86 × 10^−5^	2.15 (1.48–3.13)	0.19
Recessive	5.29 × 10^−1^	1.17 (0.72–1.92)	0.25

*OR, Odds ratio; CI, confidence interval; SE, standard error*.

## Discussion

In this case-control study the genotype frequency of rs12979860 located in the first intron of the *IFNL4* gene on chromosome 19q13.2 and its association with VN were analyzed and a significantly higher frequency of the T-allele was detected in VN patients compared to healthy subjects. Increased ORs (additive model: OR = 1.51, dominant model: OR = 2.15) were obtained in the calculation of both the additive and the dominant models. However, the main limitation of the study is the small size of the case sample, which could lead to the observed deviation from the HWE, though the high MAF of the variation (34.7% in the combined sample) is less prone to be influenced by individual genotype changes than a low MAF variation, thereby arguing in favor of an association effect. The underlying mechanisms leading to the development of VN still need to be uncovered. According to the virus hypothesis, viral pathology induced by neurotropic viruses such as HSV-1 is thought to be the most likely cause of VN (29). This hypothesis is supported by a recent GWAS in VN, in which 3 loci were detected containing genes that are also involved in viral processes (14). Furthermore, the results obtained in this candidate gene study showing an increased risk of VN for carriers of the rs12979860 T allele support the observation that this allele is associated with increased clinical severity and recurrence rates of herpes labialis in a dose dependent manner (16). In the same study, it was shown that the replication of HSV-1 is inhibited by the induction of a type III IFN response. Interestingly, reduced IFN-λ levels were found in patients with recurrent herpes labialis compared to seropositive controls without a history of recurrence in a study by (21).

The unfavorable effect of the rs12979860 T allele has been further demonstrated by an association with virus persistence (17) and a diminished response to antiviral therapy with pegylated IFN-λ and ribavirin in patients with HCV (19, 39). Remarkably, a HCV treatment study conducted by Rallón et al. (40) showed reduced IFN- λ3 levels in T allele carriers, while rs12979860-C allele carriers were reported to have higher IFN- λ3 plasma levels (41).

Other studies demonstrated a protective effect of the rs12979860 T allele, thereby representing conflicting results on the allele effect, e.g., in studies on the replication of the cytomegalovirus in patients with solid-organ transplants and allogenic stem cell transplants. The sample size in these studies, however, was very small (42, 43).

Functional consequences of the specific alleles of rs12979860 are still under exploration. In addition to a potential role in the promoter of the *IFNL3* gene due to its position in the 5' region of the gene, rs12979860 also shows a strong LD to rs368234815, a dinucleotide insertion/deletion variant within the first exon of *IFLN4*. Unlike the TT variant, which is subjected to degradation by mRNA by nonsense mediated decay, the ΔG allele induces a frameshift which enables expression of the *INFL4* gene (22). The ΔG variant correlates perfectly with the T allele of rs12979860 in Asians (*r*^2^ = 1.0) and is well correlated in Europeans (*r*^2^ > 0.9), but only moderately in Africans (*r*^2^ ~ 0.7). Thus, the ability to express IFN- λ4, which is restricted to carriers of the ΔG variant, might explain the strong association of rs12979860 with impaired HCV clearance in Europeans, while Africans exhibit a higher association with rs368234815 (22).

Though knowledge regarding the physiological function of IFN- λ4 is still sparse, significant differences to the other type III IFNs have been described. Compared to IFN- λ3, IFN- λ4 seems to act faster during acute antiviral response, but at the same time induces the expression of genes that inhibit IFN response. For example, the expression of *USP18*, which is an inhibitor of antiviral activity of IFN- λ, was elevated in liver biopsies of HCV patients who carried the ΔG allele and were thus capable of producing IFN- λ4 (28). In addition, IFN- λ4 was shown to induce the expression of *SOCS1*, an inhibitor of anti-HCV activity of IFNs. These results are supported by the investigation of another SNP in the second exon of the *IFNL4* gene. Rs117648444 causes a substitution of an amino acid, turning IFN- λ4 into a protein variant with reduced activity, named IFN- λ4-S70. Compared with the fully active IFN- λ4-P70 variant, humans encoding the impaired IFN- λ4-S70 variant display better spontaneous HCV clearance rates and overall better treatment response rates (44). These findings suggest that the presence, rather than absence of IFN- λ4 could be the relevant disadvantage in viral defense (45). This is supported by the fact that the ΔG variant seems to be undergoing a negative selection, since 95% of Africans, but only about 54% of Europeans and 13% of Asians, carry at least one ΔG variant (23).

Besides the favorable effects of the CC genotype (rs12979860) on HCV clearance rates, this genotype was also associated with a higher rate of hepatic inflammation and fibrosis in patients with chronic HCV infection and with a higher frequency of pulmonary fibrosis in patients with systemic sclerosis, accompanied by increased IFN- λ3 activity (46, 47). A high IFN- λ3 activity thus seems to be an advantage for virus elimination, but also to mediate chronic inflammatory processes. The results of this study may therefore suggest that VN is more likely to be caused by a reactivation of HSV-1 than by inflammation in response to the virus.

In summary, the underlying pathological mechanisms for VN are not yet known, but a viral genesis is discussed, with HSV-1 being the most likely candidate (29). In an earlier study, an association of the rs12979860 T allele with the severity of recurrent herpes labialis was demonstrated (16), establishing a link to HSV-1 infection. Since the T allele of rs12979860 and the functional ΔG variant rs368234815 are in strong LD, the expression of IFN- λ4, which depends on the presence of the ΔG variant, could contribute to the differing antiviral responses between IFN- λ4 and other type III IFNs. However, in addition to the established impact of IFN- λ4 on HCV-infection further studies are needed to confirm whether an influence on HSV-1 infections also occurs. This would further strengthen the viral hypothesis of the latent HSV-1 infection for VN. Although the present study comprises a relatively small sample size and replication is necessary, these results are consistent with previous studies on rs12979860.

## Data Availability Statement

The authors acknowledge that the data presented in this study is available upon request.

## Ethics Statement

The studies involving human participants were reviewed and approved by Ethics Committee of the Ludwig-Maximilians-University, Munich, Germany. The patients/participants provided their written informed consent to participate in this study.

## Author Contributions

DR, MS, and IG were involved in the conception and design of study. MH, AH, and SM participated in the acquisition and analysis of the data. BK was responsible for the statistical analysis. All authors contributed to the article and approved the submitted version.

## Conflict of Interest

MS is Joint Chief Editor of the Journal of Neurology, Editor in Chief of Frontiers of Neuro-otology and Section Editor of F1000. He has received speaker's honoraria from Abbott, Actelion, Auris Medical, Biogen, Eisai, GSK, Heel, Henning Pharma, Interacoustics, MSD, Otometrics, Pierre-Fabre, TEVA, UCB. He is a shareholder of IntraBio and acts as a consultant for Abbott, Actelion, Heel, IntraBio, and Sensorion. The remaining authors declare that the research was conducted in the absence of any commercial or financial relationships that could be construed as a potential conflict of interest.

## References

[1] Sekitani T Imate Y Noguchi T Inokuma T Vestibular neuronitis: epidemiological survey by questionnaire in Japan. Acta Otolaryngol Suppl. (1993) 503:9–12. 10.3109/00016489309128061 8470507

[2] Adamec I Krbot Skoric M Handzic J Habek M. Incidence, seasonality and comorbidity in vestibular neuritis. Neurol Sci. (2015) 36:91–5. 10.1007/s10072-014-1912-4 25085434

[3] Huppert D Strupp M Theil D Glaser M Brandt T. Low recurrence rate of vestibular neuritis: a long-term follow-up. Neurology. (2006) 67:1870–1. 10.1212/01.wnl.0000244473.84246.76 17130428

[4] Kim YH Kim K-S Kim KJ Choi H Choi J-S Hwang IK. Recurrence of vertigo in patients with vestibular neuritis. Acta Otolaryngol. (2011) 131:1172–7. 10.3109/00016489.2011.593551 21728751

[5] Furuta Y Takasu T Fukuda S Inuyama Y Sato KC Nagashima K. Latent herpes simplex virus type 1 in human vestibular ganglia. Acta Otolaryngol Suppl. (1993) 503:85–9. 10.3109/00016489309128081 8385871

[6] Arbusow V Schulz P Strupp M Dieterich M Reinhardstoettner A von Rauch E. Distribution of herpes simplex virus type 1 in human geniculate and vestibular ganglia: implications for vestibular neuritis. Ann Neurol. (1999) 46:416–9. 10.1002/1531-8249(199909)46:3<416::AID-ANA20>3.0.CO;2-W 10482275

[7] Theil D Arbusow V Derfuss T Strupp M Pfeiffer M Mascolo A. Prevalence of HSV-1 LAT in human trigeminal, geniculate, and vestibular ganglia and its implication for cranial nerve syndromes. Brain Pathol. (2001) 11:408–13. 10.1111/j.1750-3639.2001.tb00408.x 11556685 PMC8098601

[8] Himmelein S Lindemann A Sinicina I Horn AKE Brandt T Strupp M. Differential involvement during latent herpes simplex virus 1 infection of the superior and inferior divisions of the vestibular ganglia: implications for vestibular neuritis. J Virol. (2017) 91:e00331-17. 10.1128/JVI.00331-17 28446678 PMC5487553

[9] Esaki S Goshima F Kimura H Ikeda S Katsumi S Kabaya K. Auditory and vestibular defects induced by experimental labyrinthitis following herpes simplex virus in mice. Acta Otolaryngol. (2011) 131:684–91. 10.3109/00016489.2010.546808 21526906

[10] Milionis HJ Mittari V Exarchakos G Kalaitzidis R Skevas AT Elisaf MS. Lipoprotein (a) and acute-phase response in patients with vestibular neuronitis. Eur J Clin Invest. (2003) 33:1045–50. 10.1111/j.1365-2362.2003.01275.x 14636287

[11] Kassner SS Schöttler S Bonaterra GA Stern-Straeter J Hormann K Kinscherf R. Proinflammatory activation of peripheral blood mononuclear cells in patients with vestibular neuritis. Audiol Neurootol. (2011) 16:242–7. 10.1159/000320839 20980744

[12] Michel NA Zirlik A Wolf D. CD40L and its receptors in atherothrombosis-an update. Front Cardiovasc Med. (2017) 4:40. 10.3389/fcvm.2017.00040 28676852 PMC5477003

[13] Giegling I Hosak L Mössner R Serretti A Bellivier F Claes S. Genetics of schizophrenia: A consensus paper of the WFSBP Task Force on Genetics. World J Biol Psychiatry. (2017) 18:492–505. 10.1080/15622975.2016.1268715 28112043

[14] Rujescu D Hartmann AM Giegling I Konte B Herrling M Himmelein S. Genome-wide association study in vestibular neuritis: involvement of the host factor for HSV-1 replication. Front Neurol. (2018) 9:591. 10.3389/fneur.2018.00591 30079052 PMC6062961

[15] Greco A Macri GF Gallo A Fusconi M Virgilio A de Pagliuca G. Is vestibular neuritis an immune related vestibular neuropathy inducing vertigo? J Immunol Res. (2014) 2014:459048. 10.1155/2014/459048 24741601 PMC3987789

[16] Griffiths SJ Koegl M Boutell C Zenner HL Crump CM Pica F. A systematic analysis of host factors reveals a Med23-interferon-lambda regulatory axis against herpes simplex virus type 1 replication. PLoS Pathog. (2013) 9:e1003514. 10.1371/journal.ppat.1003514 23950709 PMC3738494

[17] Duggal P Thio CL Wojcik GL Goedert JJ Mangia A Latanich R. Genome-wide association study of spontaneous resolution of hepatitis C virus infection: data from multiple cohorts. Ann Intern Med. (2013) 158:235–45. 10.7326/0003-4819-158-4-201302190-00003 23420232 PMC3638215

[18] Dill MT Duong FHT Vogt JE Bibert S Bochud P-Y Terracciano L. Interferon-induced gene expression is a stronger predictor of treatment response than IL28B genotype in patients with hepatitis C. Gastroenterology. (2011) 140:1021–31. 10.1053/j.gastro.2010.11.039 21111740

[19] Tanaka Y Nishida N Sugiyama M Kurosaki M Matsuura K Sakamoto N. Genome-wide association of IL28B with response to pegylated interferon-alpha and ribavirin therapy for chronic hepatitis C. Nat Genet. (2009) 41:1105–9. 10.1038/ng.449 19749757

[20] Murakawa M Asahina Y Nakagawa M Sakamoto N Nitta S Kusano-Kitazume A. Impaired induction of interleukin 28B and expression of interferon lambda 4 associated with nonresponse to interferon-based therapy in chronic hepatitis C. J Gastroenterol Hepatol. (2015) 30:1075–84. 10.1111/jgh.12902 25611696

[21] Pica F Volpi A Gaziano R Garaci E. Interferon-lambda in immunocompetent individuals with a history of recurrent herpes labialis. Antivir Ther. (2010) 15:737–43. 10.3851/IMP1610 20710055

[22] Prokunina-Olsson L Muchmore B Tang W Pfeiffer RM Park H Dickensheets H. A variant upstream of IFNL3 (IL28B) creating a new interferon gene IFNL4 is associated with impaired clearance of hepatitis C virus. Nat Genet. (2013) 45:164–71. 10.1038/ng.2521 23291588 PMC3793390

[23] O'Brien TR Prokunina-Olsson L Donnelly RP. IFN-lambda4: the paradoxical new member of the interferon lambda family. J Interferon Cytokine Res. (2014) 34:829–38. 10.1089/jir.2013.0136 24786669 PMC4217005

[24] Syedbasha M Egli A. Interferon lambda: modulating immunity in infectious diseases. Front Immunol. (2017) 8:119. 10.3389/fimmu.2017.00119 28293236 PMC5328987

[25] Lopušná K ReŽuchová I Kabát P Kúdelová M. Interferon lambda induces antiviral response to herpes simplex virus 1 infection. Acta Virol. (2014) 58:325–32. 10.4149/av_2014_03_325 25518713

[26] Ank N West H Bartholdy C Eriksson K Thomsen AR Paludan SR. Lambda interferon (IFN-lambda), a type III IFN, is induced by viruses and IFNs and displays potent antiviral activity against select virus infections in vivo. J Virol. (2006) 80:4501–9. 10.1128/JVI.80.9.4501-4509.2006 16611910 PMC1472004

[27] Marcello T Grakoui A Barba-Spaeth G Machlin ES Kotenko SV MacDonald MR. Interferons alpha and lambda inhibit hepatitis C virus replication with distinct signal transduction and gene regulation kinetics. Gastroenterology. (2006) 131:1887–98. 10.1053/j.gastro.2006.09.052 17087946

[28] Obajemu AA Rao N Dilley KA Vargas JM Sheikh F Donnelly RP. IFN-λ4 attenuates antiviral responses by enhancing negative regulation of IFN signaling. J Immunol. (2017) 199:3808–20. 10.4049/jimmunol.1700807 29070670 PMC5698113

[29] Strupp M Magnusson M. Acute unilateral vestibulopathy. Neurol Clin. (2015) 33:669–85. 10.1016/j.ncl.2015.04.012 26231279

[30] Saber Tehrani AS Kattah JC Kerber KA Gold DR Zee DS Urrutia VC. Diagnosing stroke in acute dizziness and vertigo: pitfalls and pearls. Stroke. (2018) 49:788–95. 10.1161/STROKEAHA.117.016979 29459396 PMC5829023

[31] First MB Spitzer RL Gibbon M Williams JBW Structured Clinical Interview for DSM-IV Axis I Disorders, Clinician Version (SCID-CV). Washington, DC: American Psychiatric Press, Inc (1996).

[32] First M Gibbon M Spitzer R Williams J Benjamin L. Structured Clinical Interview for DSM-IV Axis II Personality Disorders, (SCID-II). Washington, DC: American Psychiatric Press, Inc. (1997).

[33] Rice JP Reich T Bucholz KK Neuman RJ Fishman R Rochberg N. Comparison of direct interview and family history diagnoses of alcohol dependence. Alcohol Clin Exp Res. (1995) 19:1018–23. 10.1111/j.1530-0277.1995.tb00983.x 7485811

[34] Oeth P del Mistro G Marnellos G Shi T van den Boom D. Qualitative and quantitative genotyping using single base primer extension coupled with matrix-assisted laser desorption/ionization time-of-flight mass spectrometry (MassARRAY). Methods Mol Biol. (2009) 578:307–43. 10.1007/978-1-60327-411-1_20 19768603

[35] 1000 Genomes Project Phase 3. Available online at: http://www.ensembl.org. rs12979860 1000 Genomes Project Phase 3 allele frequencies. (accessed June 5, 2018).

[36] Purcell S Neale B Todd-Brown K Thomas L Ferreira MA Bender D. PLINK: a tool set for whole-genome association and population-based linkage analyses. Am J Hum Genet. (2007) 81:559–75. 10.1086/519795 17701901 PMC1950838

[37] Purcell SM Chang C PLINK 1.7. Available online at: http://pngu.mgh.harvard.edu/purcell/plink/ (accessed February 28, 2018).

[38] Gauderman WJ. Sample size requirements for association studies of gene-gene interaction. Am J Epidemiol. (2002) 155:478–84. 10.1093/aje/155.5.478 11867360

[39] Ge D Fellay J Thompson AJ Simon JS Shianna KV Urban TJ. Genetic variation in IL28B predicts hepatitis C treatment-induced viral clearance. Nature. (2009) 461:399–401. 10.1038/nature08309 19684573

[40] Rallón NI Soriano V Naggie S Restrepo C McHutchison J Vispo E. Impact of IL28B gene polymorphisms on interferon-λ3 plasma levels during pegylated interferon-α/ribavirin therapy for chronic hepatitis C in patients coinfected with HIV. J Antimicrob Chemother. (2012) 67:1246–9. 10.1093/jac/dkr598 22294646 PMC3695611

[41] Langhans B Kupfer B Braunschweiger I Arndt S Schulte W Nischalke HD. Interferon-lambda serum levels in hepatitis C. J Hepatol. (2011) 54:859–65. 10.1016/j.jhep.2010.08.020 21145813

[42] Bravo D Solano C Giménez E Remigia MJ Corrales I Amat P. Effect of the IL28B Rs12979860 C/T polymorphism on the incidence and features of active cytomegalovirus infection in allogeneic stem cell transplant patients. J Med Virol. (2014) 86:838–44. 10.1002/jmv.23865 24374819

[43] Egli A Levin A Santer DM Joyce M O'Shea D Thomas BS. Immunomodulatory function of Interleukin 28B during primary infection with cytomegalovirus. J Infect Dis. (2014) 210:717–27. 10.1093/infdis/jiu144 24620020

[44] Terczynska-Dyla E Bibert S Duong FHT Krol I Jorgensen S Collinet E. Reduced IFNlambda4 activity is associated with improved HCV clearance and reduced expression of interferon-stimulated genes. Nat Commun. (2014) 5:5699. 10.1038/ncomms6699 25534433

[45] Wack A Terczynska-Dyla E Hartmann R. Guarding the frontiers: the biology of type III interferons. Nat Immunol. (2015) 16:802–9. 10.1038/ni.3212 26194286 PMC7096991

[46] Eslam M McLeod D Kelaeng KS Mangia A Berg T Thabet K. IFN-λ3, not IFN-λ4, likely mediates IFNL3-IFNL4 haplotype-dependent hepatic inflammation and fibrosis. Nat Genet. (2017) 49:795–800. 10.1038/ng.3836 28394349

[47] Metwally M Thabet K Bayoumi A Nikpour M Stevens W Sahhar J. IFNL3 genotype is associated with pulmonary fibrosis in patients with systemic sclerosis. Sci Rep. (2019) 9:14834. 10.1038/s41598-019-50709-9 31619697 PMC6795812

